# Overcoming barriers to resident scholarly productivity and research at a large academic institution

**DOI:** 10.15694/mep.2019.000213.1

**Published:** 2019-11-26

**Authors:** Michael Dennis, Felipe Batalini, Lindsay Demers, Ashish Upadhyay

**Affiliations:** 1 Boston Medical Center and Boston University School of Medicine; 2 Beth Israel Deaconess Medical Center and Harvard Medical School

**Keywords:** scholarship, research, resident, barriers, internal medicine

## Abstract

This article was migrated. The article was marked as recommended.

**Background:** Scholarship is an important component of resident education that can increase future opportunities in academic medicine. Each year the Accreditation Council for Graduate Medical Education surveys internal medicine (IM) residents for their satisfaction with scholarship opportunities. The IM residents at our large academic center have consistently reported scores that were lower than expected. We designed this study to identify barriers to resident scholarship and successful interventions.

**Objective:** Identify barriers to resident scholarly productivity and report the results of several interventions aimed at improving resident scholarship.

**Methods:** Leaders within the IM residency program were interviewed with a standardized questionnaire, and an online survey was distributed to IM residents. Comparisons were made between program leader interviews and resident survey responses.

**Results:** Program leaders unanimously agreed there are abundant research opportunities, that resident research is prioritized, and that time is the major research barrier. Conversely, only 72% of residents reported satisfaction with research opportunities, 56% felt that the program prioritized research, and finding a mentor was the most frequently reported research barrier (60%). Residents considered early discussions about research the most successful intervention to improve scholarship.

**Conclusion:** Finding a mentor was the most important barrier to resident scholarship and discussing research early in the intern year was the most successful intervention.

## Introduction

Medical trainees are keenly attuned to the value of scholarly work. It is well recognized as a determining factor for residency programs, fellowship opportunities, and academic positions ( Alguire *et al*., 1996; Bertram *et al*., 2015; Green, Jones and Thomas, 2009; McGaghie, 2009; Mikhail and Bernstein, 2007). Medical students and resident physicians alike have a vested interest in programs with high levels of scholarship, as this is often the gateway to the next phase of their career. Their perception of such opportunities may influence their willingness to attend individual academic centers ( Chan and Peterson, 2015; Stillman *et al*., 2016). Furthermore, the Accreditation Council for Graduate Medical Education (ACGME) now requires internal medicine (IM) residency programs to ensure that all residents participate in scholarly activity ( ACGME, 2018). It is therefore in each institution’s best interest to foster a high level of scholarship.

The IM residency program at Boston University School of Medicine and Boston Medical Center (BMC) is a strong University-based program linked to a large urban tertiary-care hospital. The program has consistently produced a high level of scholarly work by the IM house staff and faculty, and annual funds from the National Institute of Health rank the institution in the top 10% nationally ( National Institute of Health, 2018). Despite these accomplishments, annual surveys from the ACGME show that residents express dissatisfaction in their ability to be easily involved in scholarly projects.

In an effort to understand and improve residents’ perceptions of available scholarship opportunities, we designed a mixed-methods study to first interview program leaders within the IM residency program and then survey IM residents to identify knowledge gaps or potential barriers to scholarship. We hypothesized that residents’ perspectives would differ from program leaders in a meaningful way and that these differences may explain the lower than expected ACGME scores on domains pertaining to scholarship. We anticipate that other institutions may find themselves below the national average on ACGME surveys and that a similar approach to the one presented herein may help identify previously unrecognized barriers to scholarly productivity. At the very least, we believe the interventions recommended in our study will be broadly applicable to other institutions that are seeking to improve resident scholarship.

## Methods

### Key Informant Interviews

Three key informants who held leadership positions in the department of medicine were interviewed. These semi-structured interviews were conducted by the same investigator between June and August 2018. All interviews were recorded and then manually transcribed by two of the co-authors. A list of interview questions can be found in Supplemental Table 1.

### Resident Questionnaire

A sixteen-question, web-based survey was created to gauge resident opinions on the research opportunities and scholarly productivity at BMC (Supplemental Table 2). The survey asked about residents’ research experience during residency, including the number of research projects, the ability to find a research mentor, barriers to starting and completing research, and helpful resources. Results from the key informant interviews were taken into account, with a focus on the barriers and resources available for scholarly work. The survey was emailed to all internal medicine residents in the first, second, and third year of training (n = 148). Forty-nine residents responded for a response rate of 33%. Six records were excluded from analysis because they did not provide answers to the questions of interest.

## Results/Analysis

### Key informant interviews

Key informants were asked about resident scholarship in regards to the following 8 domains: (1) perception of research opportunities, (2) resident scholarly productivity, (3) barriers to residents conducting research, (4) barriers to faculty conducting research with residents, (5) research disparities across medicine specialties, (6) prioritization of research by program leadership, (7) attempts to increase scholarly activity, and (8) future goals. Table 1 lists themes that were identified within these domains along with representative quotes.

**Table 1.** Key informant interviews

Domain	Theme	Response
Perception of research opportunities	Abundant research opportunities	They have an almost unlimited array of options because there are twice as many faculty members funded to do research as the number of internal medicine residents.
Resident scholarly productivity	Opportunity for growth	I am actually impressed with the amount of scholarly productivity. [But] I think it can always be better.
Barriers to residents conducting research	Time limitation	The schedule limits dedicated research time... Our 3+1 system gives residents small chunks of elective time. We cannot give consecutive months of elective, which would be very helpful for people interested in basic science research.
	Faculty/ resident relationship	I find it really difficult to bring people together.
	Funding	We do not fund research.
	Motivation	I think there is a mixed bag of motivation in residents.
Barriers to faculty conducting research with residents	Time limitation	The time required. Faculty are under productivity pressures.
	Past experiences	If the resident behaves badly it can contaminate the future aspirations of the faculty and get them discouraged.
	Funding	It is an unfunded effort. Residents, although they are very talented, may not have enough time to work within the goals of funding.
Research disparities across medicine specialties	Unequal distribution of opportunities	Yes...not all of our sections have robust research programs. Some are extremely robust, and some not at all robust.
	Unequal distribution of resident interest	Yes, there are disparities, not particularly because of the specialty itself, but because the demand is different. There are five research sections that residents rarely work with.
Research prioritized by program leaders	Resident research opportunities are a priority	Yes, they are a very high priority. We have an obligation to expose people to careers in discovery.
Attempts to increase scholarly activity	Chair of Medicine involvement - successful intervention	The chair of medicine has been personally meeting with residents early in the PGY-2 year...There are fewer people coming and saying I can’t find a mentor. I am hoping that represents progress.
	Scholarship committee - successful intervention	The establishment of research committees where residents actually lead this effort has been a positive influence.
	Discuss research early in intern year - successful intervention	We decided to introduce research at the intern retreat. We made them [interns] think about research early...showed them the resources... and asked them to talk about it with their faculty advisor by the end of the year. I think that made them think about research early on and not wait until the end of the intern year. We should probably continue doing that. I think those efforts have been successful.
	Research database - neutral intervention	We established a research database, [where] we approach faculty every year and ask them if there are projects that they want resident involvement in. We collect that information and put it on the website as a database. I found that it has mixed reviews.
	Assigning mentors - failed intervention	We tried assigning mentors to residents. It does not work, because it is like blind dates. There has to be chemistry.
	Research nights - failed intervention	We tried to have research nights where we would have mentors come and meet with residents. The residents said that they wanted [these], but they would never show up for the meetings.
Future goals	Improving resident and faculty connections	We need to make it easier for residents to connect with faculty. There are way more landing spots for residents than there are residents.
	Advertising	We should be doing more advertising.
	Chief resident dedicated to improving scholarship	For the first time, we have a chief resident with a focus on scholarship.
	Increase funding	We will be applying for the NIH R38 grant this year, which would give us resources and money to involve people in an extended research experience during residency.

PGY = post-graduate year, NIH = National Institutes of Health

Interviewees were asked to describe their perception of the research opportunities available to IM residents. All three respondents described an abundance of research opportunities. One interviewee said, “they have an almost unlimited array of options,” noting “there are twice as many faculty members funded to do research as the number of internal medicine residents.” Another mentioned the strength of funding in the program to back resident research projects. *Perception of research opportunities*

### Resident scholarly productivity

Interviewees were asked where they think the IM program stands in terms of resident scholarly productivity. Two of the three respondents thought there was an *opportunity for growth* One respondent said, “I am actually impressed with the amount of scholarly productivity. [But] I think it can always be better.” The words “impressed” and “proud” were also mentioned. One of the respondents said, “I think we do extremely well in terms of scholarly productivity.” Notably, this statement came from one of the respondents that thought there was still room for growth.

### Barriers to residents conducting research

Interviewees were asked to identify major barriers that prevented IM residents from conducting research. The one barrier mentioned by all three respondents was *time limitation.* Interviewees frequently cited the resident schedule as a major barrier. One respondent said, “the schedule limits dedicated research time.. our 3+1 system gives residents small chunks of elective time” (3 +1 refers to three weeks of ward or elective followed by one week of ambulatory block scheduling). The respondent also referred to the fact that the current schedule requires an ambulatory week every month, preventing “consecutive months of elective, which would be very helpful for people interested in basic science research.” A second respondent stated, “I worry that we do not have adequate blocks of time that can be linked together.”

### Barriers to faculty conducting research with residents

Interviewees were asked to identify the major barriers that prevented faculty from participating in resident scholarly projects. The most predominant theme mentioned was time limitations. Pressure from external sources regarding productivity, funding constraints, and mentoring experience were all cited as explanations for why faculty members might feel there is an inadequate amount of time to dedicate to resident projects.

*Past experiences* were also noted to negatively affect the faculty’s willingness to participate in resident projects. One respondent said, “If a resident behaves badly it can contaminate the future aspirations of the faculty and get them discouraged.” There were also reports of faculty members “spending a lot of time trying to mentor residents doing research, and the resident would just disappear.” It is easy to imagine why a faculty member may not be enthusiastic to engage in resident scholarly projects after such experiences.

### Research disparities across medicine specialties

Interviewees were asked if there are disparities in resident research opportunities across medical specialties. All respondents agreed that there are disparities, and two themes emerged. The first was deemed the *unequal distribution of opportunities.* Two of the respondents referenced this theme, stating “not all of our sections have robust research programs” and “some are extremely robust, and some not at all robust.”

The second theme was called the *unequal distribution of resident interest.* The gastroenterology, cardiology, and oncology sections were specifically cited as high demand research specialties. Fellowships in these fields were considered “competitive” and resident scholarly projects were considered a necessity for anyone considering entry to these fields. Conversely, there were five specialties within the Department of Medicine where residents were noted to rarely pursue scholarly work.

### Prioritization of research by program leadership

Interviewees were asked whether resident research opportunities were prioritized by leaders in the IM program. All respondents said yes, resident research is a priority, and two of the three responded emphatically with strong statements. Excerpt from a respondent: “yes, it is a very high priority. We have an obligation to expose people to careers in discovery.”

### Strategies to increase scholarly activity

Interviewees were asked to identify past strategies to increase resident scholarship. The successful interventions were (1) the *involvement of the chair of Medicine,* (2) the establishment of a *scholarship committee,* and (3) *discussing research early in the intern year.* The establishment of a *research database,* where an online list is compiled of ongoing faculty projects was considered a neutral intervention. Two unsuccessful interventions were *assigning mentors* and *“research nights*”. Regarding the former strategy, one respondent noted, “assigning mentors does not work, because it is like a blind date. There has to be chemistry.” *Research nights* were considered unsuccessful because there was a poor showing of residents to these events.

### Future goals for research

The final question asked respondents to comment on the current strategy to increase scholarship. All three respondents had different answers to this question. One respondent said “we need to make it easier for residents to connect with faculty. There are way more landing spots for residents than there are residents.” Another said, “we should be doing more advertising.” Having a chief resident with a dedicated goal of improving research and improving the amount of research money were also mentioned.

### Resident survey

An electronic survey was distributed to all 148 IM residents at BMC. Forty-three participants (29%) completed ≥ 90% of the survey questions ( Table 2). The majority of residents indicated that they will be pursuing a medicine subspecialty fellowship (91%). The mean number of research projects was 2.6 (standard deviation 2.0, median 3, range 0-8), and the number of research projects did not differ significantly as residents progressed through PGYs. The minority of respondents thought it was easy to find a research project (37%), but 72% thought there were satisfactory research opportunities in their field of interest. Forty percent of respondents had a goal of leading their own research project, and of that 40%, 35% had a difficult time finding a mentor that was willing to support this goal. Fifty-six percent of residents felt supported by their research mentors. This feeling of support increased significantly as residents progressed through PGY (F=5.0, df = 2, p = 0.01). Fifty-six percent of residents also felt they had sufficient guidance. There was an even split in terms of who the residents thought provided the most helpful guidance, 42% responded faculty member and 42% responded resident (16% responded “other person”). Only 56% of respondents felt that the program prioritized research.

**Table 2.** Internal medicine resident survey

	All Residents (n=43)	PGY-1 (n=13)	PGY-2 (n=13)	PGY-3 (n=17)
Pursuing Fellowship, %	91	100	100	76
Research Projects, median (range)	3 (0-8)	1 (0-8)	3 (0-7)	3 (0-6)
Easy to find a research project, %	37	31	39	41
Satisfactory research opportunities in the field of interest, %	72	62	62	88
Goal of leading their own research project, % Difficult to find a mentor willing to support this goal ^a^	40 35	31 25	54 29	35 50
Felt supported, %	56	31	46	82
Satisfactory guidance finding research projects, %	59	64	62	53
Whose guidance was most helpful in finding research project(s), % Faculty member Resident Other	42 42 16	54 31 15	31 46 23	41 47 12
Felt the program prioritized research, %	56	46	69	53
Found the following resources to be helpful, % Discussing research early in intern year Research database Chair of medicine meeting with residents ^b^ Resident scholarship committee Research mixers with faculty and residents	77 26 23 21 15	92 31 NA 8 23	73 28 9 9 18	67 20 33 40 7

^a^ Percent calculated based on the number of residents that had this goal;

^b^ Chair of medicine did not meet with PGY-1 residents; PGY = post-graduate year

Residents were also asked to identify barriers that prevented them from starting or completing research projects. As shown in Table 3, the inability to find a research mentor and time were the two main barriers cited by residents for not starting or completing a research project. Interventions that were considered helpful are also listed in Table 2. The majority of respondents thought it was helpful to discuss research early in the intern year (77%). Other resources were reported as helpful by a minority of respondents: research database (26%), chair of medicine meeting with PGY-2 residents (23%), resident-led scholarship committee (21%), and research mixers with faculty and residents (15%).

**Table 3.** Research barriers

	Starting a Project	Completing a Project
Inability to find a mentor	60%	n/a
Lack of time	58%	79%
Lack of research training	28%	37%
Unsure of area of interest/Lack of interest	23%	13%
Too few opportunities in specific area of interest	14%	n/a
Lack of funding	7%	5%

## Discussion

In this study, we identified major barriers to resident scholarship and examined the perceived success of several interventions to improve scholarly activity. Perhaps the most notable finding was the validation of dyssynchronous perspectives between the IM residents and program leaders regarding scholarship opportunities. We found that program leaders unanimously thought research was a high priority, while just over half the surveyed residents reported feeling this way (56%). This finding suggests a possible communication gap between the program leadership and residents with regard to the importance of resident research. Program leaders also unanimously agreed there were abundant research opportunities within the specialty and subspecialties of IM, yet only 37% of residents thought it was easy to find a research project. This discrepancy raised the question of whether there truly was a lack of research opportunities unbeknownst to the program leaders or if residents simply struggle to find available research projects and lack the perception of all the opportunities. This finding provided a potential explanation for the lower than expected ACGME survey results and suggested that an improvement in this area may bolster satisfaction scores on subsequent ACGME surveys.

The two groups also disagreed on the major research barriers. For example, finding a mentor was the most frequently identified barrier for residents starting a research project (60%); this was only mentioned by one program leader. The importance of this finding cannot be understated, as prior publications have always identified time as the most frequently reported barrier by residents and program leaders ( Gill *et al*., 2001; Nair *et al*., 2019; Levine, Hebert and Wright, 2005). In our study, time was the second most frequently identified barrier (58%). This suggested that finding a mentor is a significant problem at our institution and that program leaders may not be fully aware of its significance. Additional results from the resident survey provided further validation of this problem, as only 59% of residents thought there was satisfactory guidance for finding a project. We hypothesize that the current culture in many academic hospitals may unintentionally enforce this barrier, as residents these days have less clinical encounters with faculty who predominantly engage in research. Primary ward teaching is now largely relegated to hospitalists or clinical educators whose involvement has improved clinical teaching but could have hampered the opportunities for connections between residents and research faculty. Additional efforts are thus necessary to connect residents with faculty members actively involved in research.

Notably, several interventions have been implemented at our institution that could improve resident opinions of scholarship opportunities. We created a scholarship committee of IM residents, coordinated research mixers with faculty and residents, established a research database listing available projects and mentors, formally discussed research early in the intern year, and the chair of medicine has been meeting with PGY-2 residents to help identify mentors. Of these interventions, resident and faculty social events, the research database, and one-on-one meetings with program leaders have been tried with some degree of success at other institutions ( Rothberg *et al*., 2014). However, IM residents at BMC most frequently listed discussing research early in the intern year as helpful (77%), while all the other interventions were considered helpful by 26% of respondents or less. In contrast, the Chair of Medicine involvement and the scholarship committee were most often reported as successful by program leaders. Only one program leader thought discussing research early in the intern year was successful. This again highlighted the disconnect between the perceptions of IM residents and program leaders. Nevertheless, we believe any intervention that is considered helpful may be worth pursuing, even if it benefits a minority of residents.

There were also notable similarities between the two groups. For example, program leaders and residents recognized an opportunity for growth, identified time as a major limitation, and acknowledged that research opportunities are not equal across all subspecialties. They also identified several major barriers to resident scholarship, including time, funding, motivation, and established relationships between residents and faculty members. Similar results have been reported in prior publications ( Levine, Hebert and Wright, 2005; Alguire *et al*., 1996; Gill *et al*., 2001; Rivera, Levine and Wright, 2005).

Two major findings came out of our study. First, we were able to clearly demonstrate the disconnect between residents’ and program leaders’ perceptions regarding scholarly activity. This finding has broad applicability to other programs that are looking to improve the amount of resident scholarly work. We would advise program leaders to facilitate resident input on research barriers so that funds and efforts can be directed to the areas of greatest need. We also identified mentorship as a major barrier to scholarship. Although we have tried several interventions to improve mentor identification, only discussing research early in the intern year was listed as helpful by the majority of residents. We would, therefore, advise all programs to facilitate these discussions at the beginning of residency, perhaps as early as intern orientation for those programs that have a structured introductory period. We also note that 42% of residents thought their fellow residents were helpful in identifying research mentors. Therefore, efforts aimed at encouraging resident-to-resident research discussions might be successful.

We acknowledge the fact that our study was conducted at a single institution with a relatively small sample size, but nevertheless believe these findings will be relevant to the majority of programs that are looking to improve resident scholarship. Other limitations included the low resident survey response rate (28%), the unclear definition of scholarship, which could be defined broadly as any academic achievement or more traditionally as research resulting in peer-reviewed publication, and the high percent of survey respondents pursuing fellowship (91% compared to the previously reported rate of 65% obtained from a large national survey) ( West and Dupras, 2012). Notably, the high percentage of survey respondents pursuing fellowship indicates some degree of selection bias, and these residents may have higher expectations for scholarly opportunities than the residents that are not pursuing a fellowship.

## Conclusion

In summary, we were able to demonstrate dyssynchronous perspectives between IM residents and program leaders regarding scholarship opportunities. Several important research barriers were identified, as were multiple interventions that have had varying degrees of success. Finding a mentor was the most important barrier to resident scholarship and discussing research early in the intern year was the most successful intervention. These findings highlight unmet needs within the IM residency program and offer a way forward towards increased scholarly productivity and higher satisfaction ratings on the annual ACGME surveys. We hope that our results herein provide a framework to identify research barriers and successful interventions for institutions that are seeking to improve resident scholarship.

## Take Home Messages

• Finding a mentor was the most frequently reported barrier to starting a research project
• Discussions about research early in the intern year were considered helpful by the majority of residents
• Program leaders may not be aware of the major barriers to resident scholarship and the success of interventions aimed at improving scholarship opportunities
• Feedback from the house staff within residency programs can identify barriers to scholarly work that program leaders are unaware of; this input can then be used to allocate time and funding to the areas with the greatest need

## Notes On Contributors

**Michael Dennis**, MD, is an internal medicine resident at Boston Medical Center, Boston, Massachusetts, USA. ORCID ID: https://orcid.org/0000-0002-8175-5311

**Felipe Batalini**, MD, is a hematology and oncology fellow at Beth Israel Deaconess Medical Center and Harvard Medical School, Boston, Massachusetts, USA. ORCID ID: https://orcid.org/0000-0003-3354-308X

**Lindsay Demers**, MS, PhD is an assistant professor in the Department of Medicine at Boston University School of Medicine and Boston Medical Center, Boston, Massachusetts, USA.

**Ashish Upadhyay**, MD is an associate professor in the Department of Medicine at Boston University School of Medicine and Boston Medical Center, Boston, Massachusetts, USA. ORCID ID: https://orcid.org/0000-0002-0536-5776
