# Supplementary material for: Overcoming barriers to resident scholarly productivity and research at a large academic institution
Source: MedEdPublish (2016). 2019 Nov 26;8:213. [Version 1] doi: 10.15694/mep.2019.000213.1 (PMC10712469; doi:10.15694/mep.2019.000213.1)
Supplement: Peer review history

# Reviewer report: Reviewer response for version 1

**DOI:** 10.21956/mep.19942.r30274

**Published:** 2020-03-12

**Authors:** Ken Masters

**Affiliations:** 1 Sultan Qaboos University

This review has been migrated. The reviewer awarded 3 stars out of 5 An interesting paper about barriers to resident scholarly productivity and research at a large academic institution. Overall the study appears to have been done very well, with useful information gathered from both staff and students. While the exact figures might differ with those in other institutions, it is quite possible that similar disparities exist in other institutions, and so this paper serves as a useful warning for those institutions to conduct a similar exercise.Some issues:• The title of the paper (“Overcoming barriers to resident…”.) is too broad, and, as a result, is misleading. The paper does not describe how to *overcome* the barriers; rather, it *identifies* the barriers. So, the paper needs to be re-titled to “Identifying barriers….” (This aligns with the objective, as given in the paper).• The questions in the interviews are somewhat problematic:a. The description of the interviews is a little short. Looking at the interview questions, I have to wonder if these were the only questions put to the interviewees. If they were, then they could have been done through a simple interview form. If, however, there are further elaborations and discussions, then this should be made a little clearer in the description of the methodology.b. In addition, one of the limitations of any questionnaire is that one gets answers only to the questions one asks. Interviews, however, allow the interviewee to roam further than narrow questions, and take the process into areas not envisaged by the researcher. Many of these questions are very focused (two are even Yes/No questions), so it really would have been better if the questions had been far more open-ended. (Again, if this was done in the actual interview, then this should be described in the paper).It is too late to change the questions now, but I would like to see a little more elaboration and discussion of these issues in the paper.• The authors should give an indication of any software used (for both the quantitative and qualitative analysis, testing and theming)• For the quantitative data reporting, raw numbers plus percentages (not only percentages) should be given.• The last portion of the last item in the take-home message (“this input can then be used to allocate time and funding to the areas with the greatest Need”) is speculation or suggestion, and is not supported by the results in the paper, so should be removed.So, the paper does have quite a bit of strength, but does need some tightening. I look forward to a Version 2 of the paper that addresses these concerns.

Reviewer Expertise:

NA

No decision status is available

# Reviewer report: Reviewer response for version 1

**DOI:** 10.21956/mep.19942.r30273

**Published:** 2019-12-04

**Authors:** P Ravi Shankar

**Affiliations:** 1 American International Medical University

This review has been migrated. The reviewer awarded 4 stars out of 5 I enjoyed reading this paper about resident research in the Internal Medicine program at a premier academic institution. The dichotomy in perceptions in many areas between faculty leaders and residents has been highlighted. Many residents at the point of entry to the program may not have much idea about research and possible research topics as has been mentioned by the authors. The reasons why residents find it difficult to find faculty research mentors have also been briefly discussed. The authors have also discussed the limitations of their study. The low response rate among residents could be a major limitation. I am not sure if the self-selected respondents represent the overall resident population which has also been briefly mentioned by the authors. This article will be of special interest to residency program directors.

Reviewer Expertise:

NA

No decision status is available
